# High Plasma Glucagon Levels Correlate with Waist-to-Hip Ratio, Suprailiac Skinfold Thickness, and Deep Subcutaneous Abdominal and Intraperitoneal Adipose Tissue Depots in Nonobese Asian Indian Males with Type 2 Diabetes in North India

**DOI:** 10.1155/2017/2376016

**Published:** 2017-05-28

**Authors:** Shajith Anoop, Anoop Misra, Surya Prakash Bhatt, Seema Gulati, Harsh Mahajan, Gokulraj Prabakaran

**Affiliations:** ^1^Center of Nutrition & Metabolic Research (C-NET), National Diabetes, Obesity and Cholesterol Foundation (N-DOC), Safdarjung Development Area, New Delhi, India; ^2^Diabetes Foundation (India), Safdarjung Development Area, New Delhi, India; ^3^Fortis C-DOC Centre of Excellence for Diabetes, Metabolic Diseases and Endocrinology, Chirag Enclave, Nehru Place, New Delhi, India; ^4^Mahajan Imaging Centre, Safdarjung Development Area, New Delhi, India

## Abstract

We aimed to correlate plasma glucagon levels with anthropometric measures and abdominal adipose tissue depots. Nonobese males (*n* = 81; BMI < 25 kg/m^2^) with T2DM of less than one-year duration and nonobese males without diabetes (*n* = 30) were evaluated for the following: anthropometry (BMI, waist circumference, W-HR, and truncal skinfolds), whole-body DEXA (for body fat and fat-free mass), and MRI scan (for volumes of subcutaneous abdominal adipose tissue (SCAT) including superficial and deep, intra-abdominal visceral adipose tissue (including intraperitoneal adipose tissue (IPAT), retroperitoneal adipose tissue, liver span and fatty liver, and pancreatic volume)). Plasma glucose and glucagon, serum insulin, hepatic transaminases, and lipid profile were measured. Significantly higher levels of fasting and postprandial glucagon (*p* < 0.001) and fasting and postprandial insulin (*p* < 0.001) were seen in patients with T2DM. The mean values of fasting and postprandial plasma glucagon levels were higher in T2DM patients with NAFLD (*n* = 37) as compared to T2DM patients without NAFLD (*n* = 44). Four independent predictors were derived for fasting glucagon levels in patients with T2DM, namely, W-HR, suprailiac skinfold thickness, IPAT, and deep SCAT (*p* < 0.05; *r*^2^ = 0.84). These observations in Asian Indians may have significance for diabetes therapies which impact glucagon levels.

## 1. Introduction

Obesity, an important etiological factor for metabolic syndrome and type 2 diabetes (T2DM), is increasing in the Indian subcontinent [1]. While obesity is the main contributor to diabetes in India, nearly 20–30% of patients have body mass index (BMI) < 25 kg/m^2^ [2], thus belonging to nonobese category. In such patients, we have recently demonstrated increased intra-abdominal adipose tissue volume (IAAT) and increased hepatic fat as compared to nondiabetic individuals of similar body weight and BMI [3, 4]. While such increased abdominal adiposity in “nonobese” patients contributes to insulin resistance and metabolic syndrome, the extent of hyperglucagonaemia and its correlates is unclear in Asian Indians with abdominal obesity and excess hepatic fat.

Glucagon is a 29 amino acid polypeptide (molecular weight of 3485 daltons) and a nonsteroid hormone, produced by *α* cells of the pancreas. Endogenous glucagon helps in maintaining euglycaemia by stimulating glucose production via hepatic glycogenolysis and gluconeogenesis when plasma glucose declines to subnormal levels. Pancreatic glucagon is also secreted during food ingestion thereby regulating satiety signals leading to termination of food intake [5, 6]. In T2DM, the functions of pancreatic *α* cells are increased resulting in hyperglucagonaemia, which causes increased hepatic glucose production, and hyperglycaemia [7]. In patients with diabetes, the secretory response of *α* cells to low-glucose concentrations is impaired predisposing to risk of hypoglycaemia, especially in patients treated with insulin [8].

Research studies on adiposity and diabetes in Asian Indians have been done primarily in context of insulin secretion and insulin resistance, [9, 10] whereas plasma glucagon and its relation to adiposity in this ethnic group remain sparsely researched. In the present study on young, nonobese (BMI < 25 kg/m^2^) Asian Indians with T2DM, we intend to describe correlations of plasma glucagon levels in relation to anthropometry, volumes of abdominal adipose tissue depots, liver span, nonalcoholic fatty liver disease (NAFLD), and pancreatic volume.

## 2. Methodology

Ethical approval was obtained from the institutional review board, and the study was conducted according to the Declaration of Helsinki 2013 [11].The details of study subjects have been mentioned in our previous study [3, 4]. Briefly, nonobese (BMI < 25 kg/m^2^) male patients with T2DM (cases, *n* = 81), aged between 18 and 40 years, and diagnosed within one year from onset of diabetes and nonobese, nondiabetic males (*n* = 30) were recruited. None of the patients was on insulin therapy, sulphonylureas, dipeptidyl peptidase (DPP4) inhibitors, glucagon-like peptide-1 (GLP-1) analogues, pioglitzone, or any antihyperglycaemic treatment other than metformin.

Waist circumference was measured midway between the iliac crest and the lower-most margin of the ribs while individual was standing erect, and hip circumference was measured at the maximum circumference of the gluteal muscles. The mean of three readings of each circumference was taken for the calculation of the waist-to-hip ratio. Truncal skinfolds [subscapular, supra iliac, and abdominal skinfolds (horizontal and vertical)] and sum of all truncal skinfolds were measured using a Lange skinfold calliper (Beta Technology Inc., Santa Cruz, CA, USA) as reported previously [12].

Whole-body dual-energy X-ray absorptiometry (DEXA) imaging was done to estimate total body fat percentage, fat mass, fat-free mass, and lean muscle mass in the truncal region, arms, and legs. Further, magnetic resonance imaging (MRI, 1.5 Tesla) was done using T1-weighted axial scans at lumbar vertebra 2 and 3 to estimate the volumes of total abdominal and abdominal adipose tissue compartments, namely, subcutaneous abdominal adipose tissue (SCAT; anterior, posterior, superficial, and deep) and intra-abdominal adipose tissue [IAAT; intraperitoneal (IPAT) and retroperitoneal (RPAT)], liver span, and pancreatic volume, using previously published protocols [3].

The definition of NAFLD was based on the presence of hepatic steatosis on imaging in the absence of significant alcohol intake, viral infections, and use of hepatotoxic drugs [13]. In this study, the size of the liver was measured at the level of the fifth intercostal space at the mid-clavicular plane using fast spoiled gradient echo (FSPGR) sequence. Two images (one in-phase and one out-of-phase) were obtained for each slice. Hepatic fat infiltration was estimated by measuring the signal differences between the two images and graded as normal, grade 1, grade 2, or grade 3 NAFLD [14]. Pancreatic volume was measured using 3D liver acquisition with volume acceleration (LAVA) pulse sequence as previously [3]. Pancreatic volume index was calculated as pancreatic volume (cm^3^)/body surface area (m^2^) [15].

### 2.1. Biochemical Analysis

Fasting and postprandial blood samples, after a standard meal of 265 calories (nutrient composition: proteins: 11.9%, carbohydrates: 77.2%, and fats: 10.6%) were analysed for glycaemic parameters, lipid profile, and hepatic transaminases as mentioned previously [3]. For glucagon analysis, blood samples were collected in EDTA vaccutainers treated with aprotinin (Trasylol 10000 KIE/ml; Bayer HealthCare AG 51368) which was added to give a final concentration of 200 KIE per ml of peripheral blood. Plasma glucagon levels were measured by radioimmunoassay method using commercially available kits (Glucagon RIA kit, Millipore, 6 Research Park Drive, St. Charles, Missouri 63304, USA). The interassay and intra-assay coefficients were 1.50 and 3.28, respectively. Plasma insulin levels were measured by enzyme-linked immunosorbent assay (ELISA) using commercial kits (USCN, Life Sciences Inc., Houston, TX 77082, USA). For serum insulin, the interassay and intra-assay percentage coefficient variables were 1.76 and 2.10, respectively.

### 2.2. Statistical Analysis

Data were analysed using STATA 11.0 (College Station, Texas, USA), and continous variables were presented as mean ± standard deviation. The correlations of fasting plasma glucagon with anthropometry, measures of body composition on DEXA, abdominal adipose tissue compartments, pancreatic volume, liver span, and biochemical variables on MRI were assessed using Pearson's correlation. Stepwise multiple linear regression analysis was applied to derive determinants of fasting plasma glucagon levels. The *p* value less than 0.05 was considered statistically significant.

## 3. Results

**Figure 1:**
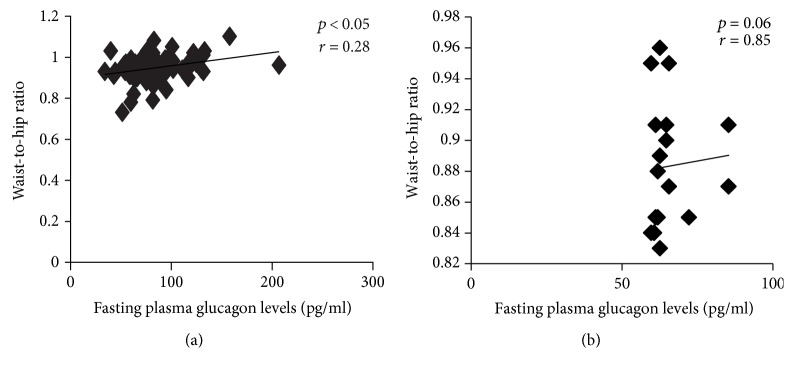
Correlation of fasting plasma glucagon levels with waist-to-hip ratio in nonobese males with T2DM (cases; *n* = 81) (a) and in nonobese males without diabetes (controls: *n* = 30) (b).

**Figure 2:**
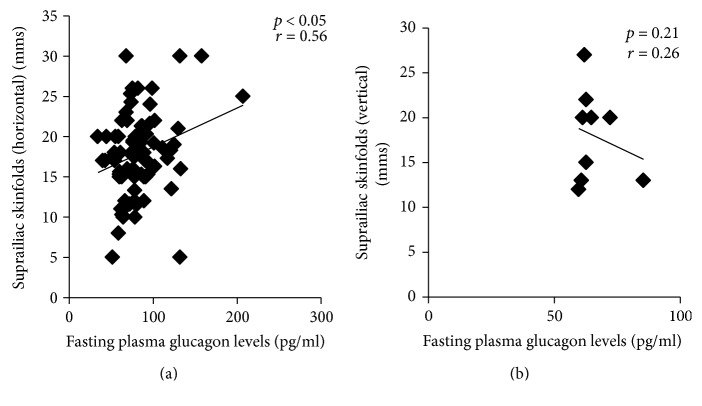
Correlation of fasting plasma glucagon levels with suprailiac skinfolds (horizontal) in nonobese males with T2DM (cases; *n* = 81) (a) and in nonobese males without diabetes (controls: *n* = 30) (b).

We observed significantly higher mean levels of fasting and postprandial insulin (*p* < 0.01), fasting glucagon (*p* < 0.01), and postprandial glucagon (*p* < 0.01) in patients with T2DM as compared with controls (Table 1). The mean values of fasting (85.9 ± 31.9 pg/ml) and postprandial glucagon levels (84.6 ± 29.0 pg/ml) were higher in T2DM patients with NAFLD (*n* = 37) as compared to T2DM patients without NAFLD (*n* = 44) (fasting: 81.0 ± 22.7 pg/ml; postprandial: 75.9 ± 19.8 pg/ml, *p* < 0.05). Pearson's correlation analysis revealed significant positive correlation of fasting plasma glucagon levels with waist circumference (*p* < 0.05) (Figures 1(a) and 1(b)), suprailiac skinfolds (*p* < 0.05) (Figures 2(a) and 2(b)), and total truncal skinfolds (*p* < 0.05) in cases as compared to controls. Among DEXA-derived measures, fasting plasma glucagon levels correlated positively with total body fat % (*p* < 0.01), truncal fat % (*p* < 0.05), and truncal fat mass (*p* < 0.05), while no significant correlation was observed with total fat-free mass.

**Figure 3:**
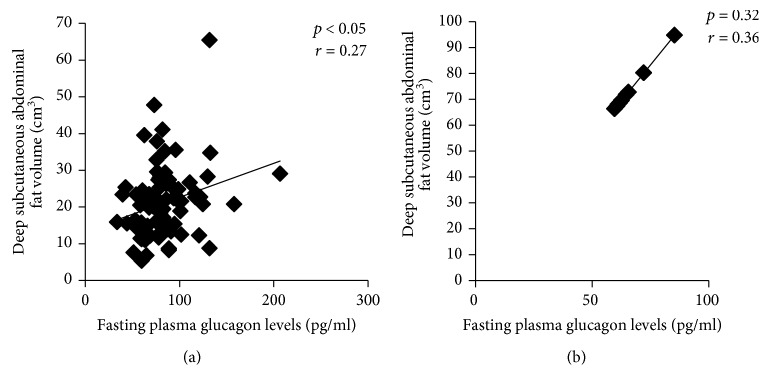
Correlation of fasting plasma glucagon levels with deep subcutaneous abdominal adipose tissue volume in nonobese males with T2DM (cases; *n* = 81) (a) and in nonobese males without diabetes (controls: *n* = 30) (b).

**Figure 4:**
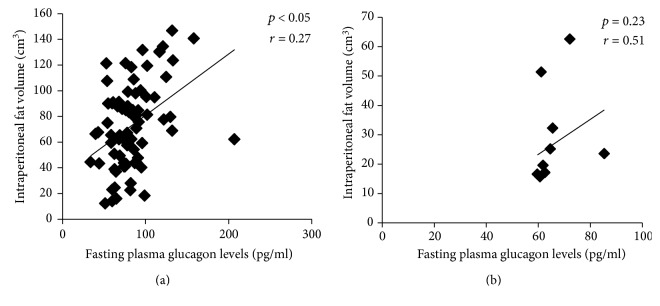
Correlation of fasting plasma glucagon levels with intraperitoneal adipose tissue volume in nonobese males with T2DM (cases; *n* = 81) (a) and in nonobese males without diabetes (controls: *n* = 30) (b).

On MRI, significant positive correlations were observed for fasting plasma glucagon levels with volumes of total abdominal adipose tissue volume (*p* < 0.05), total SCAT (*p* < 0.05), anterior SCAT (*p* < 0.05), posterior SCAT (*p* < 0.05), superficial SCAT (*p* < 0.05), and deep SCAT (*p* < 0.05), in cases as compared to controls (Figures 3(a) and 3(b)). For IAAT, significant positive correlation was observed for fasting plasma glucagon levels with volumes of total IAAT (*p* < 0.05) and IPAT (*p* < 0.05) (Figures 4(a) and 4(b)).

Importantly, no significant correlation was observed for fasting plasma glucagon levels with pancreatic volume, pancreatic volume index, liver span, and grades of NAFLD in T2DM patients. For biochemical variables, fasting plasma glucagon levels correlated positively (*p* < 0.05) only with low-density lipoprotein cholesterol (LDL-c) levels. However, we observed lack of significant correlation of fasting plasma glucagon levels with fasting and postprandial insulin, hepatic transaminase, and high-density lipoprotein cholesterol (HDL-c) levels in T2DM patients. No significant correlations were observed in controls for fasting plasma glucagon levels with biochemical variables (Table 2).

On stepwise multiple linear regression analysis, we derived four independent predictors for fasting plasma glucagon levels in T2DM patients, namely, waist-to-hip ratio, suprailiac skinfold thickness, deep SCAT, and IPAT (*p* < 0.05; *r*^2^ = 0.84) (Table 3). Together, all four predictors accounted for up to 84% variability in fasting plasma glucagon levels, with no multicollinearity between each of them [variance inflation factor (VIF) value < 5.0].

## 4. Discussion

This is the first study on nonobese Asian Indian males with T2DM including detailed estimation of multiple measures of body composition using skinfolds, DEXA, and MRI and shows high-fasting plasma glucagon levels and its correlation with several measures of abdominal adiposity. Specifically, we report four adiposity measures which independently predict fasting plasma glucagon levels, namely, waist-to-hip ratio, suprailiac skinfold thickness, and volumes of deep SCAT and IPAT—indicators of posterior truncal subcutaneous adipose tissue and abdominal adiposity. Importantly, patients with type 2 diabetes in this study were diagnosed within year of onset and were managed by lifestyle modifications and metformin therapy.

It is important to note that Asian Indians have more abdominal adiposity and intra-abdominal adipose tissue, accompanied by higher degrees of insulin resistance, as compared to white Caucasians [16]. Of note, South Asians with BMI in normal range (22.0 kg/m^2^) have thicker SCAT and were more insulin resistant as compared to white Caucasians in USA [10]. Previously, we have reported waist circumference, triceps skinfolds, and body fat percentage as predictors of insulin resistance in 793 healthy Asian Indian adolescents of North India [17]. The importance of truncal and abdominal subcutaneous adipose tissue was shown by us again in another study where sum of four skinfolds strongly predicted fasting hyperinsulinaemia and HOMA-IR values in Asian Indian children, with a consistent increase in fasting insulin as skinfold thickness increased [18]. Subsequently, using MRI, we reported that increased SCAT in healthy Asian Indians correlated independently to metabolic syndrome, irrespective of total body fat and intra-abdominal fat [19]. Importantly, South Asians have excess deep SCAT even at BMI < 25 kg/m^2^ as compared to white Caucasians [20]. This observation is of further importance since a nonuniform expansion of deep SCAT correlates with abdominal visceral adipose tissue and contributes to peripheral and hepatic insulin resistance and cardiovascular risk irrespective of other adiposity indices, specifically in men [21].

Observations of thicker truncal adipose tissue in Asian Indians [10, 22] assume further importance since larger subcutaneous adipocytes have been seen in them than in white Caucasians; such adipocytes being conducive to the development of insulin resistance [23]. Of further interest is that mRNA expression was significantly higher in abdominal subcutaneous adipose tissue for several genes associated with inflammation in nonobese Asian Indians as compared with white Caucasians; associated with significantly lower rates of glucose disposal including lower plasma adiponectin concentration in the former [23]. Hence, it is reasonable to assume that abdominal subcutaneous tissue is of major importance for insulin resistance and subclinical inflammation in Asian Indians. In this context, our observation of correlation of suprailiac skinfold and deep SCAT with fasting plasma glucagon levels appears potentially important.

The glucoregulatory functions of insulin have been widely studied, whereas the association of glucagon with abdominal obesity has been sparsely researched, and no study is available in South Asians. Specifically, positive correlation of plasma glucagon levels with IAAT volume, as seen in our study, can be compared to a computed tomography-based study on estimation of abdominal fat depots, wherein higher plasma glucagon and insulin levels were observed in young, obese Canadian men (30–40 years age, BMI: 28–34 kg/m^2^) with higher intra-abdominal obesity. Moreover, in this study, significant positive correlation was observed for plasma insulin and glucagon responses to adrenaline infusion, suggestive of impaired plasma glucose-insulin homeostasis due to intra-abdominal visceral obesity [24]. Finally, the role of subcutaneous and visceral abdominal adipocyte anatomy and physiology on glucagon secretion has not been adequately researched.

Physiologically, in fasting state, exogenous glucagon reduces triglycerides, cholesterol levels, and release of low-density lipoprotein cholesterol (LDL-c) via the glucagon receptor with stimulated fatty acid oxidation, leading to release of free fatty acids [25]. In contrast to the above findings, we observed significant positive correlation of endogenous plasma glucagon levels with LDL-c levels, despite statin therapy in our patients. The cause and significance of this observation are not clear; however, altered liporegulatory role of glucagon may be contributory.

Glucagon has important actions on the liver; regulation of gluconeogenesis and postprandial hepatic glucose production in a pulsatile manner and also on metabolism of amino acids [26–28] In this context, we report simple correlation of hyperglucagonaemia with hepatic steatosis, though independent correlation was not seen. Specifically, nearly 35% of subjects (*n* = 29/81) with grade 1 NAFLD had elevated plasma glucagon levels as compared to subjects (*n* = 5/81) with grade 2 NAFLD (6.1%). Previously, using ^31^P phosphorus magnetic resonance spectroscopy (MRS) in nondiabetic Asian Indians with NAFLD, we reported increased insulin resistance and deranged gluconeogenesis pathway correlating with body mass index, body fat percentage, waist circumference, and fasting serum insulin levels [29]. Importantly, T2DM subjects with higher plasma glucagon levels are predisposed to hypoaminoacidaemia, especially of amino acids involved in gluconeogenesis, such as alanine, glycine, and proline [30]. Furthermore, in patients with T2DM and fatty liver, the signal transduction for glucagon is dysfunctional in the liver leading to impaired ureagenesis and deranged amino acid levels [31]. These metabolic features need to be investigated further with regard to the unique phenotype in nonobese Asian Indians with NAFLD and T2DM. Whether therapeutic amelioration of NAFLD through low-calorie diet intervention and aerobic exercise would improve altered gluconeogenesis and have a decremental effect on elevated plasma glucagon in Asian Indians with T2DM needs to be researched.

Specifically, it is important to note elevated fasting plasma glucagon levels in nonobese patients with T2DM on metformin therapy in our study. It could be argued that such therapy may alter plasma glucagon levels. Of importance, a recent study in obese (BMI > 30 kg/m^2^) prediabetic North American subjects has reported that metformin therapy did not alter plasma glucagon levels despite lowering fasting plasma glucose, insulin, and C-peptide levels [32]. Whether high plasma glucagon levels in Asian Indians patients, related to adiposity, would respond better to DPP4 inhibitor therapy leading to more effective lowering of glycaemia as compared to other ethnic groups, remains to be researched.

In summary, we report elevated fasting plasma glucagon levels determined by several measures of subcutaneous abdominal and intra-abdominal adipose tissue in nonobese Asian Indians with T2DM. Of the four predictors of plasma glucagon, W-HR and subscapular skinfold thickness could be measured in a simple manner and can be applied in population-based studies. Intra-abdominal and subcutaneous adipose tissue volumes can be quantified in research settings where appropriate imaging facilities are available, but currently remain in the realm of research. Clinical significance of high-fasting plasma glucagon levels in patients with T2DM especially in those with excess adiposity measures needs further research.

## Figures and Tables

**Table 1:** Biochemical profile of nonobese males with T2DM (cases; *n* = 81) and nonobese males without diabetes (controls; *n* = 30).

Biochemical variables	Cases (*n* = 81)	Controls (*n* = 30)	*p* value
Fasting blood glucose (mg/dl)	147.8 ± 51.1	89.5 ± 8.3	<0.01
Postprandial blood glucose (mg/dl)	222.5 ± 82.9	89.3 ± 13.7	<0.01
Glycosylated haemoglobin (%)	9.0 ± 2.5	5.2 ± 0.37	<0.01
Fasting serum insulin (ng/ml)	9.5 ± 6.1	5.5 ± 2.6	<0.01
Postprandial serum insulin (ng/dl)	19.7 ± 5.0	8.0 ± 3.0	<0.01
Fasting plasma glucagon (pg/ml)	83.8 ± 27.1	65.6 ± 7.8	<0.01
Postprandial plasma glucagon (pg/ml)	80.2 ± 24.9	64.1 ± 8.8	<0.01
Total cholesterol (mg/dl)	175.4 ± 41.5	152.4 ± 29.1	<0.01
Serum triglycerides (mg/dl)	170.1 ± 99.4	97.1 ± 45.8	<0.01
High-density lipoprotein cholesterol (mg/dl)	40.7 ± 8.9	42.4 ± 8.3	0.31
Low-density lipoprotein cholesterol (mg/dl)	110.3 ± 31.2	96.8 ± 25.3	<0.05
Very low-density lipoprotein cholesterol (mg/dl)	33.5 ± 19.3	19.4 ± 9.1	<0.01
Serum glutamic pyruvic transaminase (U/l)	57.8 ± 21.7	45.6 ± 18.1	<0.01
Serum glutamic oxaloacetic transaminase (U/l)	27.9 ± 12.8	23.6 ± 9.2	<0.05

Values are presented as means ± SD. *p* < 0.05: statistically significant.

**Table 2:** Correlations of fasting plasma glucagon levels with measures of anthropometry, biochemical variables, body composition, and volumes of abdominal adipose tissue in nonobese males with T2DM (cases; *n* = 81) and nonobese males without diabetes (controls; *n* = 30).

Variables	Cases (*n* = 81)		Controls (*n* = 30)	
	Pearson's correlation coefficient (*r*)	*p* value	Pearson's correlation coefficient (*r*)	*p* value
Age (years)	0.03	0.74	0.63	0.07
Body mass index (kg/m^2^)	0.13	0.23	0.38	0.27
Body surface area (m^2^)	0.05	0.64	0.01	0.97
Waist circumference (cms)	0.59	**<0.05**	0.22	0.07
Hip circumference (cms)	0.03	0.75	0.65	0.08
Waist-to-hip ratio	0.28	**<0.05**	0.06	0.85
Midarm circumference (cms)	−0.04	0.74	0.10	0.77
Midthigh circumference (cms)	0.02	0.82	0.30	0.39
Bicep skinfolds (mms)	0.01	0.88	0.11	0.75
Tricep skinfolds (mms)	0.10	0.35	0.16	0.65
Thigh skinfolds (mms)	0.19	0.08	0.59	0.06
Calf skinfolds (mms)	0.09	0.41	0.01	0.95
Total peripheral skinfolds (mms)	0.13	0.24	0.29	0.40
Subscapular skinfolds	0.21	0.06	−0.08	0.82
Subscapular tricep ratio	0.10	0.33	−0.21	0.54
Suprailiac skinfolds (vertical)	0.56	**0.01**	0.21	0.28
Suprailiac skinfolds (horizontal)	0.26	**<0.05**	0.06	0.86
Average suprailiac skinfolds (mms)	0.28	**<0.05**	0.07	0.87
Total truncal skinfolds (mms)	0.22	0.05	0.01	0.97
*Biochemical variables*				
Fasting blood glucose (mg/dl)	−0.08	0.43	−0.06	0.99
Fasting insulin (ng/dl)	−0.06	0.57	−0.21	0.99
Postprandial serum glucose (mg/dl)	−0.05	0.64	0.09	0.78
Postprandial serum insulin (ng/dl)	−0.11	0.31	0.22	0.99
Glycosylated haemoglobin (%)	−0.02	0.83	−0.16	0.64
Serum glutamic oxaloacetic transaminase (U/l)	0.22	0.05	−0.22	0.47
Serum glutamic pyruvic transaminase (U/l)	0.21	0.05	−0.22	0.53
Total cholesterol (mg/dl)	0.19	0.08	0.07	0.83
High-density lipoprotein cholesterol (mg/dl)	−0.10	0.35	−0.15	0.67
Low-density lipoprotein cholesterol (mg/dl)	0.23	**0.03**	0.21	0.55
Serum triglycerides (mg/dl)	0.08	0.46	−0.13	0.71
*Measures of body composition (DEXA)*				
Total body fat %	0.24	**<0.05**	0.39	0.26
Total fat mass (kg)	0.21	0.07	0.50	0.13
Total fat-free mass (kg)	−0.10	0.39	−0.16	0.65
Total lean muscle mass (kg)	−0.10	0.39	−0.15	0.66
Total left arm fat %	0.20	0.09	0.24	0.50
Left arm fat mass (kg)	−0.11	0.95	0.15	0.66
Left arm lean muscle mass (kg)	−0.12	0.30	−0.21	0.55
Right arm fat %	0.19	0.11	0.23	0.51
Right arm fat mass (kg)	0.15	0.20	0.26	0.46
Right arm lean mass (kg)	−0.09	0.44	−0.10	0.78
Total leg fat %	0.18	0.12	0.29	0.40
Total leg fat mass (kg)	0.11	0.33	0.43	0.21
Total leg lean mass (kg)	−0.10	0.39	−0.15	0.66
Right leg fat %	0.21	0.08	0.41	0.22
Right leg lean mass (kg)	−0.08	0.47	−0.03	0.92
Truncal fat %	0.29	**<0.05**	0.53	0.10
Truncal fat mass (kg)	0.24	**<0.05**	0.53	0.11
Truncal lean mass (kg)	−0.10	0.37	−0.25	0.48
*MRI-derived abdominal adipose tissue depot* *(all volumes expressed in cm^3^ except liver span)*				
Total abdominal adipose tissue	0.31	**<0.05**	0.23	0.51
Anterior subcutaneous abdominal adipose tissue	0.24	**<0.05**	0.27	0.43
Posterior subcutaneous abdominal adipose tissue	0.22	**<0.05**	0.27	0.43
Superficial subcutaneous abdominal adipose tissue	0.23	**<0.05**	0.26	0.45
Deep subcutaneous abdominal adipose tissue	0.27	**<0.05**	0.32	0.36
Total subcutaneous abdominal adipose tissue	0.27	**<0.05**	0.28	0.43
Retroperitoneal abdominal adipose tissue fat	0.13	0.22	−0.06	0.84
Intraperitoneal abdominal adipose tissue	0.27	**<0.05**	0.23	0.51
Total intra-abdominal adipose tissue	0.25	**<0.05**	0.14	0.68
Pancreatic volume	0.06	0.57	−0.35	0.30
Pancreatic volume index	0.06	0.55	−0.34	0.32
Liver span (mm)	0.08	0.45	−0.12	0.73

*p* < 0.05: statistically significant.

**Table 3:** Stepwise multiple linear regression analysis showing predictors for fasting plasma glucagon levels in nonobese patients with T2DM (cases; *n* = 81) and nonobese, nondiabetic males (controls; *n* = 30).

Predictor variables	Standardized beta coefficient	Variance inflation factor	*p* value	Adjusted *r* square value
Waist-to-hip ratio	70.9	1.3	**<0.05**	0.84
Suprailiac skinfold thickness (horizontal)	0.58	1.9	**<0.05**	—
Deep subcutaneous abdominal adipose tissue volume (cm^3^)	0.29	1.9	**<0.05**	—
Intraperitoneal abdominal adipose tissue volume (cm^3^)	0.11	1.9	**<0.05**	—

*p* < 0.05: statistically significant.
